# Diagnosis and management of intramural ectopic pregnancy in the second trimester—a case report

**DOI:** 10.1259/bjrcr.20160095

**Published:** 2017-06-14

**Authors:** Long Kong, Ning Mao, Yinghong Shi, Heng Ma, Haizhu Xie

**Affiliations:** ^1^Department of Radiology, Jingzhou First People's Hospital Affiliated to Yangtze University, Jingzhou, China; ^2^Department of Radiology, Yantai Yuhuangding Hospital, Yantai, China

## Abstract

Intramural ectopic pregnancy is one of the rarest types of ectopic pregnancy, with risk of 1:30000. Confirmation of intramural ectopic pregnancy is difficult and is often performed intraoperatively. Intramural ectopic pregnancy often requires hysterectomy to avoid life-threatening haemorrhage. We present a case of intramural ectopic pregnancy in the second trimester, including its diagnostic criteria and treatment plan. Transvaginal ultrasound and MRI are important non-invasive methods in diagnosing this type of ectopic pregnancy. Clinicians should provide consideration to a combination of strategies and do their best to preserve patients’ uteri and fertility. In this case, clinicians excluded the gestational sac, repaired the uterus and saved the patient’s fertility.

## Background

Intramural ectopic pregnancy is a rare type of ectopic pregnancy—a pregnancy implant within the myometrium, separated from endometrial cavity and fallopian tubes or round ligament.^1^ The published articles on intramural ectopic pregnancy were usually during the first trimester, and only two published reports mention conservative surgery during the second trimester.^2,3^ However, both the uteri were ruptured. In our case, the uterus was as large as 17 weeks of gestation, which is an intramural ectopic pregnancy in the second trimester. The uterus was also not ruptured.

## Case report

**Figure 1.**
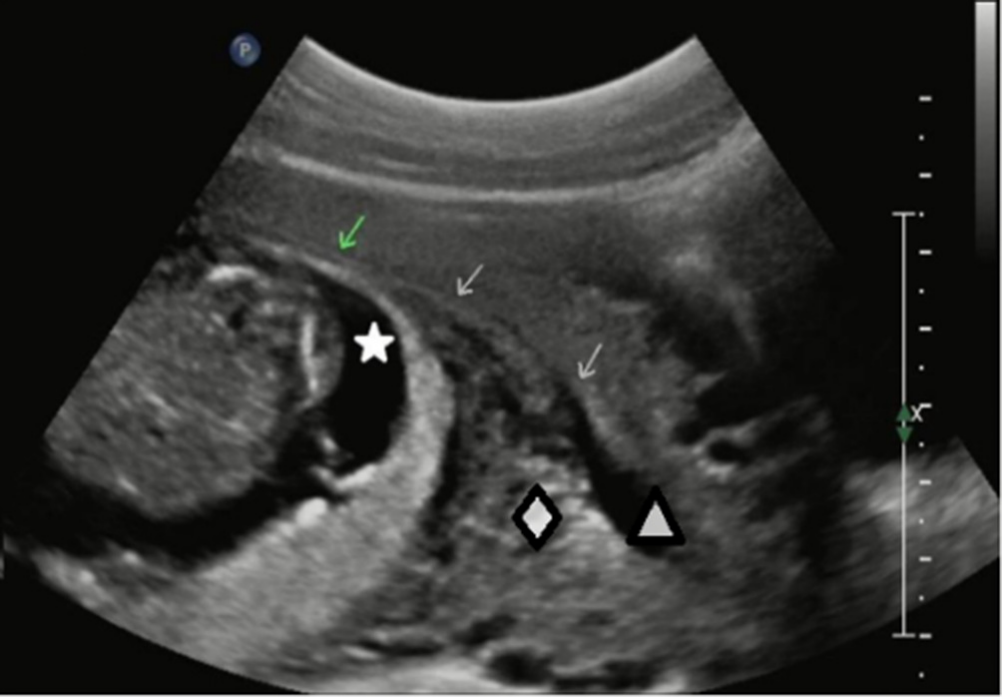
Ultrasonography showedthe gestational sac (star) at a distance from the endometrial cavity (triangle), with a compressed myometrium (diamond) between the two of them. The compressed uterus cavity distal segments (arrows) showed a cambered thin line.

**Figure 2.**
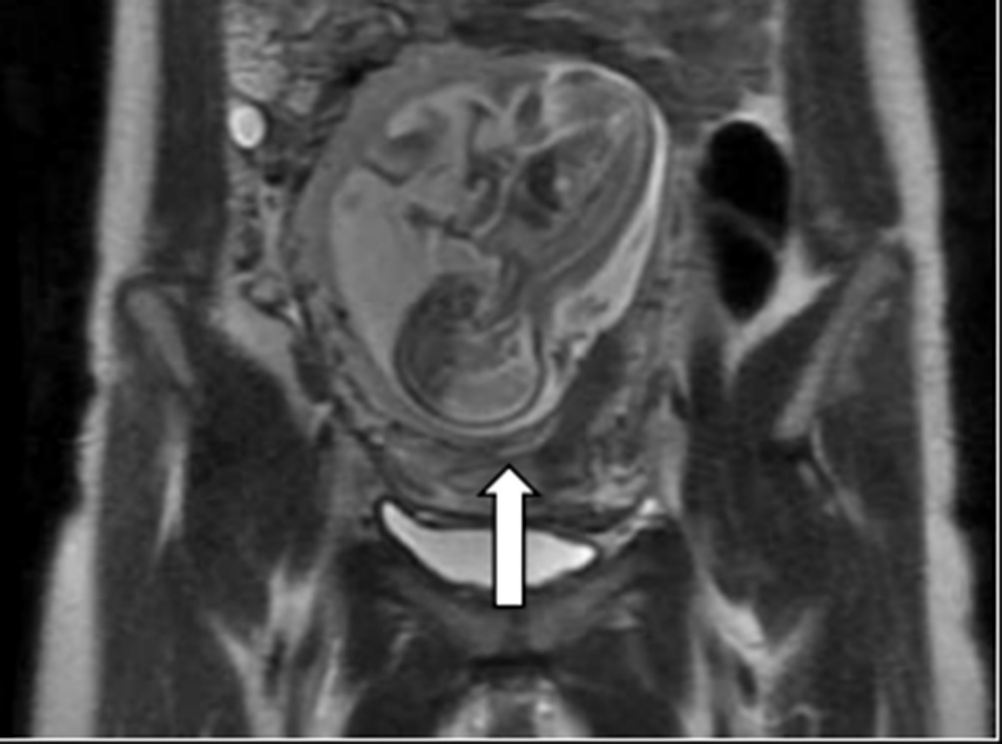
*T*_2_ weighted coronal MRI image showed a foetus with clear organs and compressed lower uterine segment (white arrow).

**Figure 3.**
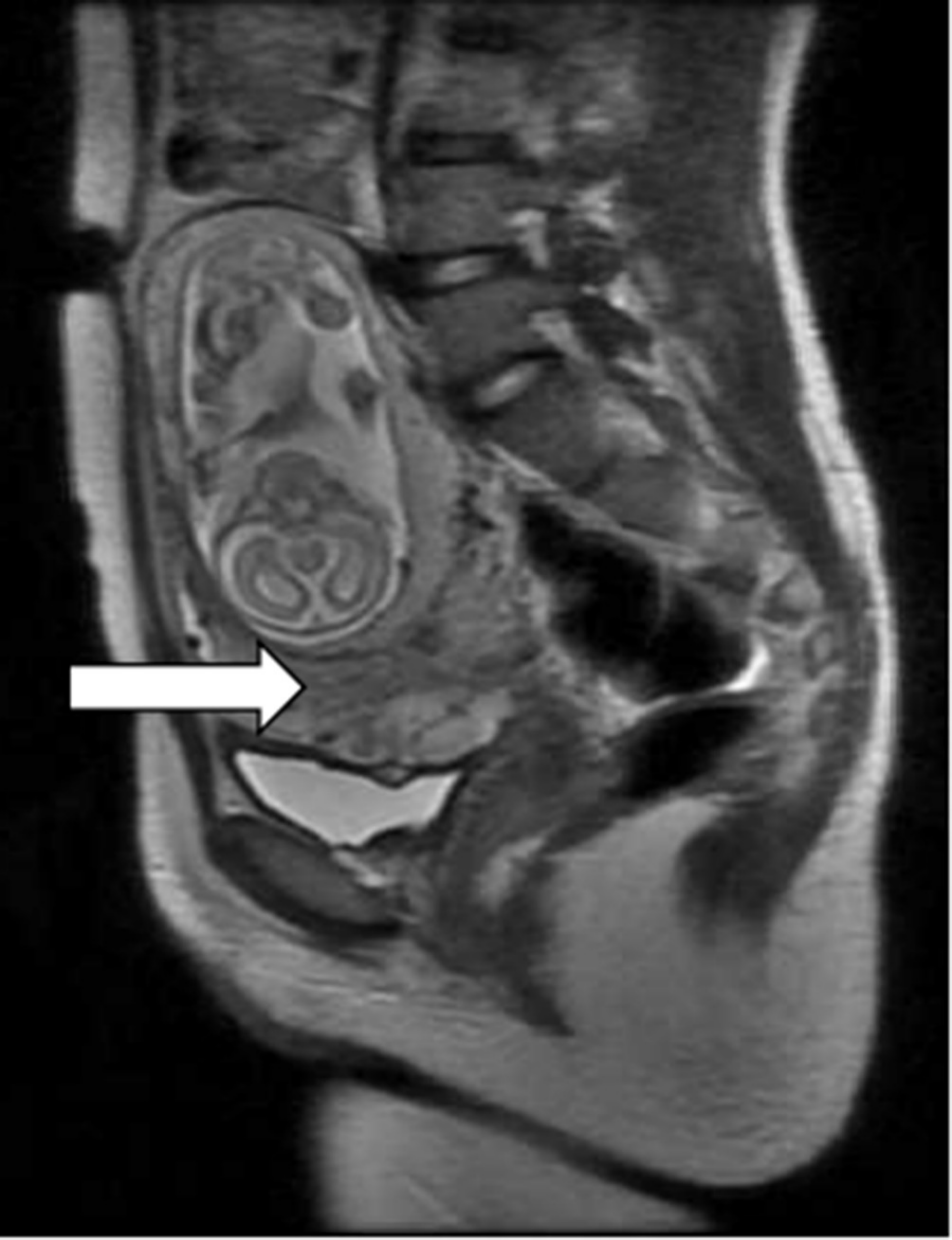
*T*_2_ weighted sagittal MRI image showed compressed lower uterine segment (white arrow).

**Figure 4.**
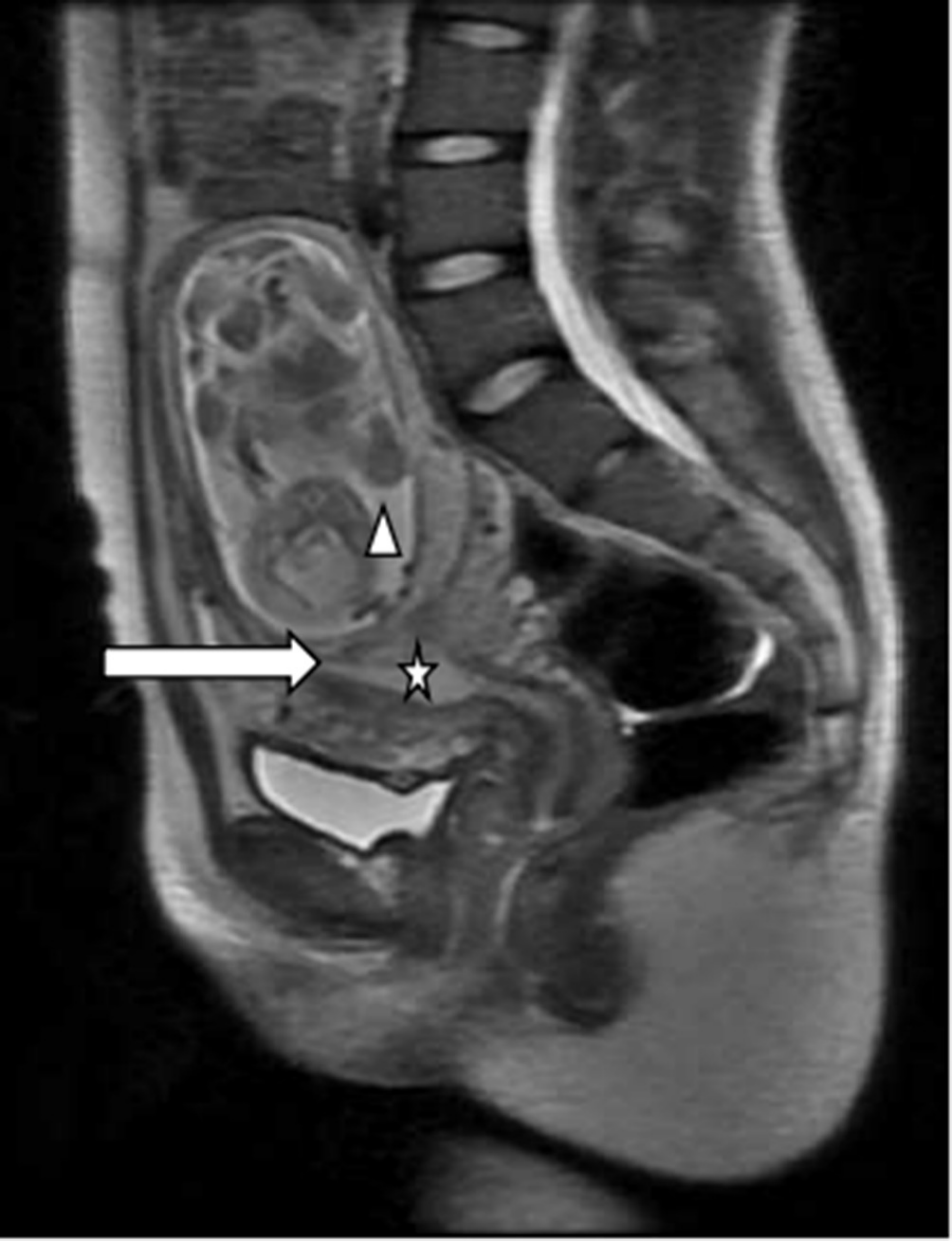
Linear hypointensity (white arrow) of the junction zone was observed between the gestational sac (triangle) and the uterine cavity (star) on *T*_2_ weighted image.

A 20-year-old asymptomatic female, G1P0, with a history of curettage, is presented to the Department of Gynecology and Obstetrics for termination of pregnancy. Her last menstrual period was 17 weeks and 2 days ago. Abdominal ultrasound revealed a clear gestational sac (GS), fetus with heartbeat and placenta previa. Abdominal ultrasound also showed the GS at a distance from the cavity, with a compressed myometrium between the two of them (Figure 1). The patient received a MRI examination, showing an enlarged uterus of 13.0 cm ×11.7 cm × 7.9 cm, because of pieces of evidence. The MRI demonstrated a foetus with clear organs (Figure 2), and compressed the lower uterine segment (Figure 3). The GS was not connected with the uterine cavity and endometrium, but embedded into the myometrium in the right posterior wall of the uterine. A linear hypointensity of the junction zone was observed between the GS and the uterine cavity on *T*_2_weighted image (Figure 4).

**Figure 5.**
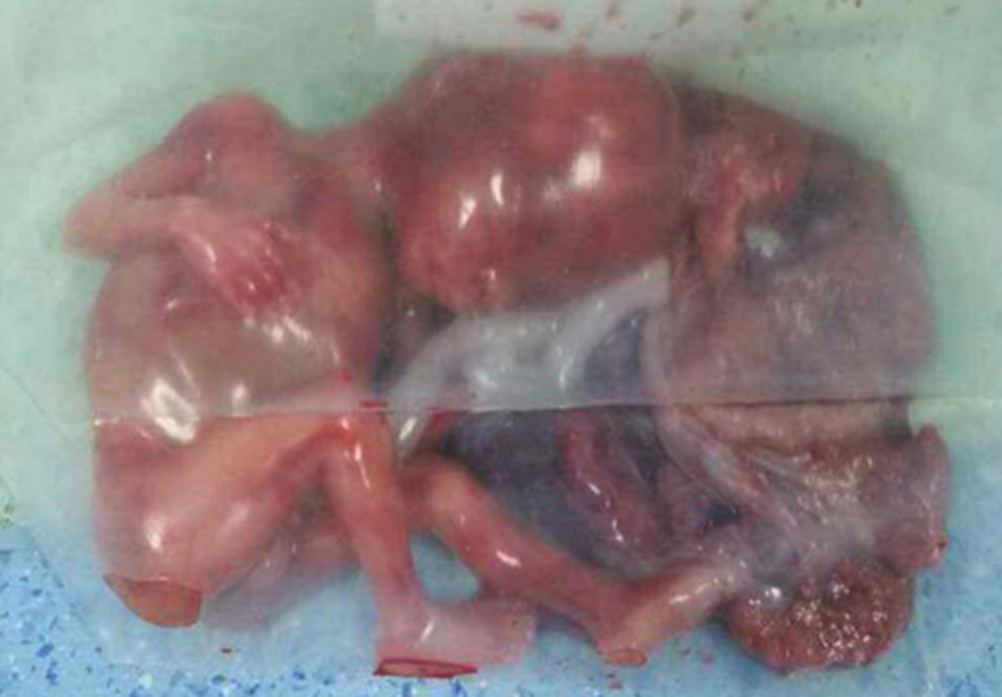
Intramural ectopic gestational sac in the second trimester was successfully excluded without life-threatening haemorrhage.

This result is likely to be placenta implantation as the myometrium cannot be separated from the placenta. The patient was at risk of the uterine rupture and life- threatening haemorrhage. Emergent management should be performed. Performing uterine artery embolization (UAE) and interventional therapy is inadvisable because of the obstructing myometrium between the cervix and placenta. Employing a surgical exploration of the abdomen was decided that was undertaken under temporary balloon occlusion of the abdominal aorta to reduce the loss of blood. The balloon was placed in the abdominal aorta between the opening of renal artery and iliac artery just before the operation. If the area of the focal damage was heavy, the subtotal hysterectomy or hysterectomy was needed. Otherwise, clinicians can perform excision by laparotomy and hysteroplasty. Given the age of patient, clinicians did their best to perform hysteroplasty instead of hysterectomy. The intramural ectopic GS in the second trimester was successfully excluded without life-threatening haemorrhage (Figure 5). The patient had an uneventful postoperative course. Her β-hCG titre decreased to 1727 mIU ml^–1^ on the second day after operation, and then to 440.1 mIU ml^–1^ on the sixth day. She was discharged 6 days after surgery.

## Discussion

Intramural ectopic pregnancy is difficult to diagnose. However, in this case, preoperative diagnosis was possible by any kinds of examination. In general, it is diagnosed by transvaginal scan (TVS) examination, hysteroscopy and confirmed by histological examination showing villous and trophoblast cells in the myometrium. In conclusion, demonstration of a live gestation embedded into the smooth muscle is the only specific sign of such pregnancy. At the same time, MRI was useful to define the relationship between the GS and the endometrium. Some *scholars* think that MRI is diagnostic gold standard of intramural pregnancy.^4^ MRI is surely “non-invasive” and useful in our case in diagnosis of intramural pregnancy. Nearly 20 published reports on intramural ectopic pregnancy were found by using the keywords: intramural ectopic pregnancy on PubMed since 1992. Only three cases were confirmed by MRI in previous published reports similar to our report. In one case, a 25-year-old female with severe abdominal pain at 18-week gestation was described by Hamouda et al,^2^ but the uterine fundus ruptured with the hemoperitoneum. Another case was a 38-year-old female at 13-week gestation. While preparing the patient for UAE, the uterus was also ruptured.^3^ A third case was a 28-year-old female with non-ruptured uterus diagnosed by MRI, for the uterus was as large as 6 weeks of gestation.^5^ In our case, the patient was in the second trimester and her uterus did not rupture, which was different from those three cases.

The management of intramural ectopic pregnancy depends on the size of the lesion, patient status and also the desire for future fertility.^6^ Verghese et al^7^ reported the successful management of an intramural pregnancy with systemic methotrexate administration.^8^ Only one case of treatment using UAE in the literature has been reported.^4^ The similarity of these two cases was that the patients were in the first trimester. Certainly, the management is individualized and combined. As seen in our case, the uterus was as large as 17 weeks of gestation which probably resulted from uterine rupture, heavy bleeding and hypovolemic shock. Hysterectomy can minimize possible complications, whereas hysteroplasty can preserve the uterus and fertility, and improve the quality of patients’ life. Given the age of patient, we performed the latter. Temporary balloon occlusion of the abdominal aorta was also used in combination with surgical enucleation to prevent intraoperative extensive bleeding. This case shows an individualized and combined management of an intramural ectopic pregnancy in the second trimester.

In conclusion, we present an unusual case of intramural ectopic pregnancy in the second trimester, including its diagnostic criteria and treatment plan. TVS and MRI are important in diagnosing this type of ectopic pregnancy. Early diagnosis and treatment can prevent serious complications such as uterine rupture and life-threatening haemorrhage.

## Learning points

1. TVS and MRI are important in diagnosing intramural ectopic pregnancy.
2. Demonstration of a live gestation embedded into the smooth muscle is the only specific sign of ectopic pregnancy
3. The management should be individualized, considerate and combined.

## Consent

Written informed consent for the case to be published (including images, case history and data) was obtained from the patient(s) for publication of this case report, including accompanying images.
